# Efficacy of a U-Shaped Automatic Electric Toothbrush in Dental Plaque Removal: A Cross-Over Randomized Controlled Trial

**DOI:** 10.3390/ijerph17134649

**Published:** 2020-06-28

**Authors:** Michele Nieri, Veronica Giuntini, Umberto Pagliaro, Monica Giani, Lorenzo Franchi, Debora Franceschi

**Affiliations:** 1 Department of Experimental and Clinical Medicine, Orthodontics, Università degli Studi di Firenze, 50127 Firenze, Italy; michelenieri@gmail.com; 2 School of Dentistry, Department of Experimental and Clinical Medicine, Università degli Studi di Firenze, 50127 Firenze, Italy; veronica.giuntini@unifi.it (V.G.); upagliaro@gmail.com (U.P.); debora.franceschi@unifi.it (D.F.); 3 Private Practice, Campi Bisenzio, 50013 Firenze, Italy; monicagiani62@gmail.com; 4 Department of Orthodontics and Pediatric Dentistry, School of Dentistry, The University of Michigan, Ann Arbor, MI 48104, USA

**Keywords:** dental plaque, powered toothbrush, randomized controlled trial, oral hygiene

## Abstract

*Background*: The aim of this single-use, four-treatment, four-period (visit), cross-over, mono-centered, examiner-blind, cross-over randomized controlled trial (RCT) was to evaluate the efficacy in dental plaque removal of a U-shaped automatic electric toothbrush (U) compared to a conventional powered toothbrush (P), a habitual toothbrushing procedure (H), and no brushing (N). *Methods*: Eligible participants were volunteer students. Primary outcome measure was the reduction in full-mouth plaque score (FMPS) after brushing. The secondary outcome variable was a visual analogic scale (VAS) on subjective clean mouth sensation. Mixed models were performed for difference in FMPS and VAS. *Results*: Twenty-two participants were randomized to the treatments in the four periods of the study. The differences between treatments in FMPS reduction after brushing were statistically significant (*p* < 0.0001). The differences were statistically significant between the U and P groups (difference −48; 95% CI from −54 to −41) favoring the P group, and between the U and H groups (difference −45; 95% CI from −52 to −39) favoring the H group. On the contrary, the difference between the U and N groups was not significant (difference 5; 95% CI from −2 to 12) favoring the U group. The differences between treatments in clean mouth VAS was statistically significant (*p* < 0.0001) favoring the P and H groups. *Conclusions*: The U-shaped automatic electric toothbrush tested in this study proved to be not effective in removing dental plaque.

## 1. Introduction

Gingivitis, periodontitis, and dental caries are the most frequent oral diseases. An important factor for their prevention is the control of pathogenic microorganisms contained in bacterial biofilm deposited on dental surfaces. This is done mainly by using a toothbrush at home [1].

Currently, the toothbrush is not only a device for oral hygiene but also an accessory that accompanies people in their common life by influencing their uses and habits [2]. Over time, many different types of manual and electric toothbrushes have been produced. A randomized trial did not reveal significant differences in bacterial plaque removal between 11 different types of manual toothbrushes [2].

On the contrary, systematic reviews have shown that some electric toothbrushes are more effective in removing bacterial plaque than manual toothbrushes [3,4]. However, all toothbrushes require a certain manual dexterity and care in use [5]. A new U-shaped automatic electric toothbrush (UAET) with silicone bristles, fully automatic and with simultaneous action on both arches, has recently been proposed. Its use could therefore be very useful in patients with psychomotor difficulties.

There are patients who are aware of how important is the home control of bacterial plaque. Low self-esteem, however, may convince them that they will not be able to achieve good results in this regard [6]. The use of an automatic toothbrush could help them to increase their self-confidence.

Some studies on the efficacy of oral hygiene motivation have shown that patient’s adherence to a program of home periodontal maintenance remains generally poor [7,8]. Additionally, in these patients, the use of an automatic toothbrush could be very useful.

However, at the moment, it is not clear how effective a UAET is in removing bacterial plaque since no randomized trial has been published on this type of toothbrush to the knowledge of the authors. Therefore, the objective of this cross-over randomized controlled trial (RCT) was to evaluate the efficacy in dental plaque removal of a UAET compared to a conventional powered toothbrush, a habitual toothbrushing procedure, and no brushing.

## 2. Materials and Methods

The experiment design followed the Consolidated Standards of Reporting Trials (CONSORT) statement [9].

### 2.1. Ethical Approval

The principles outlined in the Declaration of Helsinki on clinical research involving human subjects were adhered to. The study was approved by the ethical committee: Comitato Etico Area Vasta Centro, approval number: 15718_spe.

### 2.2. Protocol Registration

The study was registered on ClinicalTrials.gov with registration number NCT04119583 in October 2019 (https://clinicaltrials.gov/ct2/show/study/NCT04119583).

### 2.3. Trial Design

This was a single-use, four-treatment, four-period (visit), cross-over, mono-centered, examiner-blind randomized controlled trial with treatment sequences balanced for carryover effects.

There were four treatments per subject assigned in a randomized order:

- U-shaped automatic electric toothbrush (U group).
- Conventional powered toothbrush (P group).
- Habitual tooth brushing procedure (H group).
- No brushing (negative control) (N group).

### 2.4. Participants

Enrolment was proposed to 44 students of the 5th and 6th years of the Master’s Degree Course in Dentistry and Dental Prosthetics of the University of Florence, Florence, Italy. The study was illustrated during the seminars or lectures of the first semester of the 2019–2020 academic year. Thirty-one students (70%) who showed interest in participating in the study were provided with the information sheet. Eligible participants were volunteer students, aged between 18 and 30 years with presence of at least 20 teeth, no fixed orthodontic appliance, and full-mouth plaque score (FMPS) [10] at each visit above 40%.

Exclusion criteria were participants with manual disabilities to perform normal oral hygiene maneuvers and participants allergic to silicone.

All participants received a thorough explanation (V.G. and D.F.) and signed a written informed consent form prior to being enrolled in the trial. All students involved in the study had already been evaluated by the investigators in their curriculum. The study took place at the University of Florence between January and February 2020.

### 2.5. Interventions

Participants were instructed by one of the investigators (V.G.) to use at home their own toothbrush and toothpaste between each visit of the study. Participants were also instructed (V.G.) to refrain from all oral hygiene procedures and from chewing gum (for approximately 12 h) prior to their appointment time. A text message was sent to the participants the day before their appointment, as a reminder to abstain from all oral hygiene procedures and to bring all toothbrushes the next day.

At the first study visit, participants who had given signed informed consent, and who were eligible in terms of the inclusion and exclusion criteria, entered the study. Participants disclosed their plaque by means of a disclosing solution (Mira-2-Ton, Hager Werken, Duisburg, Germany), applied to the teeth with a cotton pellet. An examiner (D.F.) then performed a baseline plaque examination with a magnifying system (EyeMag Pro S 4.5X, Zeiss, Jena, Germany) using the full-mouth plaque score (FMPS) [10] on 6 sites per tooth.

Afterward, the examiner left the room, another operator (V.G.) opened an opaque and sealed envelope containing the random assigned procedure. Each subject was instructed by one of the investigators (V.G.) to brush for 2 min with the assigned toothbrush, without toothpaste, under supervision, and with the aid of a mirror. After the subject brushed their teeth, the examiner went back into the room and performed a second plaque examination. The same procedure was followed for each of the visits in turn, which were separated by an interval of at least 7 days. At each visit, participants were assigned to procedures according to their treatment sequence. Participants were assessed at each visit for their eligibility to continue in the study (FMPS above 40%).

**Figure 1:**
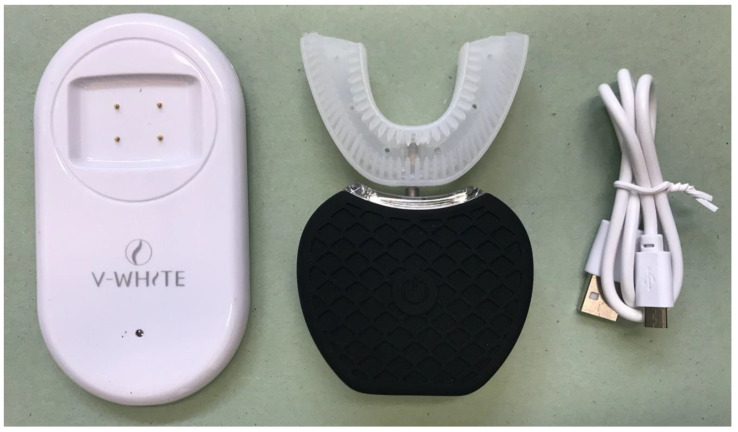
The U-shaped automatic electric toothbrush tested in this study.

There were four treatments per subject assigned in a randomized order:

- U-shaped automatic electric toothbrush (U group). The toothbrush used was the 360° Intelligent Electric Toothbrush with Cold Light; Voltage 3.2-3.7 V; Power: <5 W; Frequency 48,000 rpm/min max (Shenzhen Sure-Power Electrical Co., Ltd., Shenzhen, Guangdong, China) (Figure 1).
- Conventional powered toothbrush (P group). The toothbrush used was the Oral B Vitality CrossAction Braun (Procter & Gamble Company, Cincinnati, OH, USA).
- Habitual tooth brushing procedure (H group). Participants were asked to brush their teeth with the usual home hygiene technique (electric or manual toothbrush without toothpaste).
- No brushing (negative control) (N group). Participants were asked not to brush their teeth and wait two minutes at rest.

### 2.6. Outcomes

Primary outcome measure was the difference in FMPS between before and after brushing. The examiner (D.F.) performed the plaque assessment using a magnifying system (EyeMag Pro S 4.5X, Zeiss, Jena, Germany) and registered the presence or absence of plaque on 6 sites per tooth. The FMPS was expressed as a percentage (number of sites with plaque on the total of examined sites) (10). The examiner was preventively assessed for an intra-rater agreement measuring 738 sites two times after two hours. The kappa statistic was 0.95 (95% CI from 0.93 to 0.98).

The secondary outcome variable was a visual analogic scale (VAS) on subjective clean mouth sensation. The minimum value (0) was no clean mouth sensation, and the maximum value (10) was best sensation of clean mouth. This VAS was registered after each brushing period (V.G.) before the second plaque evaluation.

### 2.7. Sample Size

Considering a clinically relevant difference in FMPS of 15, a standard deviation of 12.90 [2], a two-tailed statistical significance threshold of α = 0.008 (Bonferroni correction), and a power of 80%, a sample size of 22 participants was necessary given an anticipated drop-out rate of 10%.

### 2.8. Randomization

The randomization list was computer generated taking into account the fact that each subject performed all 4 treatments and that the treatments were balanced within the 4 periods (visits).

The allocation sequence was concealed from the researcher (M.N.) enrolling and assessing participants in sequentially numbered, opaque, and sealed envelopes. The number on the envelope identified the patient and the visit. The envelopes were opened only when the treatment was assigned by one operator (V.G.) after the examiner (D.F.) had left the room.

### 2.9. Blinding

While the operator and patients were aware of the allocation arm, the outcome assessor was kept blinded to the allocation period.

### 2.10. Statistical Methods

Descriptive statistics were performed using mean and standard deviation. Intention-to-treat analysis was carried out.

A mixed model was performed for the difference in FMPS. In the model, the random effect was represented by the subject and the fixed effects were represented by the type of intervention (U, P, H, N), the period (1, 2, 3, 4), and the covariate represented by the FMPS registered before the brushing period. The period was added to the models only if significant. In case of statistical significance of the type of intervention, Tukey’s post hoc test was carried out.

A mixed model was implemented also for “clean mouth” sensation assessed on the VAS. In the model, the random effect was represented by the subject and the fixed effects were represented by the type of intervention (U, P, H, N) and the period (1, 2, 3, 4). The period was added to the models only if significant. In case of statistical significance of the type of intervention, Tukey’s post hoc test was performed.

Estimates for the treatment effect, *p*-values, and 95% confidence intervals were provided. The statistical software was JMP (version 13, SAS Institute Inc., Cary, NC, USA).

## 3. Results

**Figure 2:**
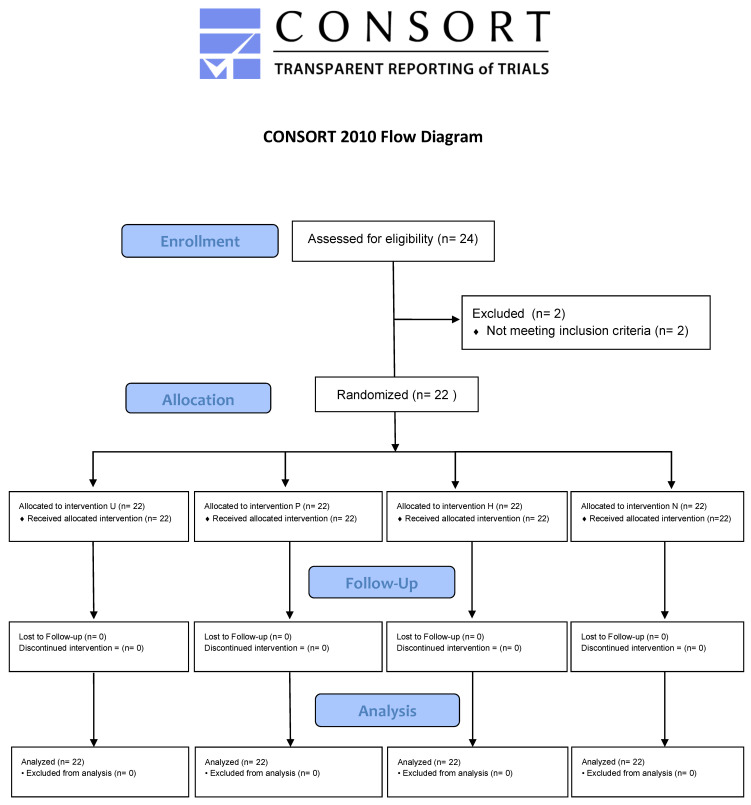
CONSORT flow diagram.

In all, 24 volunteers were sampled from the 31 students who had agreed to participate. Two students were excluded because they did not meet the inclusion criteria. Therefore, 22 participants were randomized to the treatments in the four periods of the study (Figure 2).

Participants were recruited from November to December 2019 and the study was completed by February 2020.

There were no dropouts and there were no deviations from the planned protocol.

The age of the participants was 24.4 years (SD 1.0) (min 23 years; max 27 years). There were 13 females and 9 males. Five participants were smokers.

Fifteen participants habitually used manual conventional toothbrushes and seven participants habitually used powered conventional toothbrushes.

The number of teeth was 30.3 (SD 1.5) (min 28; max 32).

The allocation of the participants to treatments per period is reported in Table 1.

The FMPS before and after the brushing period, the FMPS difference, and the clean mouth VAS for each treatment are reported in Table 2. There were no differences between treatments in FMPS before the brushing period.

The differences between treatments in FMPS reduction between before and after brushing was statistically significant (*p* < 0.0001). In particular, the difference was statistically significant between the U and P groups (difference −48; 95% CI from −54 to −41) favoring the P group, and between the U and H groups (difference −45; 95% CI from −52 to −39) favoring the H group. On the contrary, the difference between the U and N groups was not significant (difference 5; 95% CI from −2 to 12) favoring the U group. The difference between the P and H groups was also insignificant (difference 2; 95% CI from −4 to 9) favoring the P group.

The differences between treatments in clean mouth VAS was statistically significant (*p* < 0.0001). In particular, the difference was statistically significant between the U and P groups (difference −4.0; 95% CI from −5.0 to −3.0) favoring the P group, between the U and H groups (difference −3.7; 95% CI from −4.8 to −2.7) favoring the H group, and between the U and N groups (difference 2.5; 95% CI from 1.5 to 3.5) favoring the U group. The difference between the P and H groups was insignificant (difference 0.2; 95% CI from −0.8 to 1.3) favoring the P group.

## 4. Discussion

The objective of the present cross-over RCT was to compare the efficacy in dental plaque removal of a new U-shaped automatic electric toothbrush (UAET) compared to a conventional powered toothbrush, a habitual toothbrushing procedure, and no brushing. The rationale for this study was dictated by the increasing popularity of a new UAET with silicone bristles with a fully automatic and simultaneous action on both arches that has been proposed recently. Waldron et al. [5], in a recent Cochrane systematic review, emphasized that people with an intellectual disability have a higher prevalence and greater severity of periodontal disease than the general population. Moreover, their oral health deteriorates at a faster rate as they move into adulthood. Therefore, the new UAET would be very useful especially in patients with intellectual disability or in patients with motor difficulties. However, the efficacy of this new mouthpiece toothbrush had not yet been tested.

Our study showed that both a habitual toothbrushing procedure and toothbrushing with a conventional powered toothbrush were more effective in dental plaque removal when compared to either a UAET or no brushing. Habitual toothbrushing was considered as an additional active control because it represented the toothbrushing procedure that was commonly used by the participants. Powered toothbrushing was used as control because it is considered the gold standard as it is more effective than manual toothbrushing [3,4]. The duration of toothbrushing with the three procedures was standardized to 2 min. Actually, the recommended time for toothbrushing with a UAET was even shorter (45 s).

The results of this cross-over RCT clearly demonstrated that a UAET is not effective in removing dental plaque. Its efficacy was similar to no brushing. This outcome could be probably related to the fact that the silicone bristles were too short and did not reach the dental or gingival surfaces. We noticed that when the UAET was in the mouth, the silicone bristles were in many areas not in contact with either the teeth or the gingival tissues. The mouthpiece is fixed in shape and size and, therefore, it may not fit the individual dental arch shape and size.

As for the subjective clean mouth sensation, the results were similar to FMPS reduction with best scores for the P and H groups with respect to U and N.

A limitation of this study was that the four tested procedures were performed “one-shot”. No long-term effects, therefore, could be assessed. Moreover, we could not report any adverse effect that could be related to a more prolonged use of the UAET. The only outcome that was recorded was dental plaque removal. Other aspects, like gingivitis, were not assessed in the present study. Another limitation was that the study was not performed on patients but rather on a population of undergraduate students of the School of Dentistry. This aspect could limit the generalizability of the results. Future research could focus on the efficacy of the UAET in patients with psychomotor difficulties.

## 5. Conclusions

The UAET that was tested in this study proved to be not effective in removing dental plaque. In particular, the UAET was not significantly different from no brushing, while plaque removal with the UAET was significantly lower than with a powered toothbrush and a habitual toothbrushing procedure. Therefore, its use cannot be recommended for regular oral hygiene at home.

## Figures and Tables

**Table 1:** Number of participants allocated to treatment per period.

	U Group	P Group	H Group	N Group
Period 1	5	6	6	5
Period 2	6	5	5	6
Period 3	6	6	5	5
Period 4	5	5	6	6

U: U-shaped automatic electric toothbrush. P: Conventional powered toothbrush. H: Habitual tooth brushing procedure. N: No brushing (negative control).

**Table 2:** Mean full-mouth plaque score (FMPS) before and after the brushing period, FMPS difference and clean mouth visual analogic scale (VAS) for each treatment. The standard deviation is between parentheses.

	U Group *n* = 22	P Group *n* = 22	H Group *n* = 22	N Group *n* = 22	*p*-Value
FMPS before brushing	83 (11)	83 (12)	84 (13)	85 (10)	-
FMPS after brushing	72 (17)	25 (12)	28 (12)	78 (13)	<0.0001
FMPS difference	11 (8)	58 (12)	56 (12)	6 (5)	<0.0001
Clean mouth VAS	3.1 (1.8)	7.1 (1.4)	6.8 (1.4)	0.6 (0.9)	<0.0001

U: U-shaped automatic electric toothbrush. P: Conventional powered toothbrush. H: Habitual tooth brushing procedure. N: No brushing (negative control).

## References

[1] Greene J.C., Suomi J.D. (1977). Epidemiology and public health aspects of caries and periodontal disease. J. Dent. Res..

[2] Nieri M., Giani M., Pagliaro U., Picciullo A., Franceschi D., Rotundo R. (2013). Efficacy and preference of manual toothbrushes: A randomised, single blind, controlled trial. Eur. J. Oral Implantol..

[3] Yaacob M., Worthington H.V., Deacon S.A., Deery C., Walmsley A.D., Robinson P.G., Glenny A.M. (2014). Powered versus manual toothbrushing for oral health. Cochrane Database Syst. Rev..

[4] Elkerbout T.A., Slot D.E., Rosema N.A.M., Van der Weijden G.A. (2020). How effective is a powered toothbrush as compared to a manual toothbrush? A systematic review and meta-analysis of single brushing exercises. Int. J. Dent. Hyg..

[5] Waldron C., Nunn J., Mac Giolla Phadraig C., Comiskey C., Guerin S., van Harten M.T., Donnelly-Swift E., Clarke M.J. (2019). Oral hygiene interventions for people with intellectual disabilities. Cochrane Database Syst. Rev..

[6] Kneckt M.C., Keinänen-Kiukaanniemi S.M., Knuuttila M.L., Syrjälä A.M. (2001). Self-esteem as a characteristic of adherence to diabetes and dental self-care regimens. J. Clin. Periodontol..

[7] Johansson L.A., Oster B., Hamp S.E. (1984). Evaluation of cause-related periodontal therapy and compliance with maintenance care recommendations. J. Clin. Periodontol..

[8] Giani M., Pagliaro U., Franchi L., Rotundo R., Nieri M. (2019). Efficacy of four motivational techniques for improving oral hygiene. One-year follow-up of a randomized controlled trial. Clin. Trials Dent..

[9] Moher D., Hopewell S., Schulz K.F., Montori V., Gøtzsche P.C., Devereaux P.J., Elbourne D., Egger M., Altman D.G. (2010). CONSORT 2010 explanation and elaboration: Updated guidelines for reporting parallel group randomised trials. B.M.J..

[10] O’Leary T.J., Drake R.B., Naylor J.E. (1972). The plaque control record. J. Periodontol..

